# Cerebral Symptoms, Independent of Cerebral Diseases

**Published:** 1860-05

**Authors:** Charles West


### CEREBRAL SYMPTOMS INDEPENDENT OF CEREBRAL DISEASES.

BY CHARLES WEST, M. D.

In a lecture delivered at the Hospital for Sick Children, in London, Dr. West made some general remarks upon the importance of distinguishing between diseases of children depending on cerebral causes, and those which presented merely cerebral symptoms. "Do you think his brain is affected?" is a question frequently asked, and the physician's reply must be governed by his knowledge of the distinctions between true cerebral disorder and that which simulates it. Heaviness, drowsiness, a feeling of unrest, are precursory symptoms of so many affections, that it becomes necessary to detect, if possible, their real import. The younger the child, of course the more difficult will be the diagnosis. Dr. West considers three or four important points:—

1st. "The cerebral symptoms that usher in the attack, or accompany the early stages of fever." The more violent these are at first, the less likely they are to be dependent on disease of the brain; violent convulsions, for example, at this stage, being almost always connected with some morbid condition existing elsewhere. So, too, in the early stages of eruptive fevers, as measels and small-pox, convulsions of a violent nature occur, but disappear on the decided appearance of the eruption. When, however, very severe convulsions precede an attack of epidemic scarlatina, the prognosis may generally be formed of a malignant type of the disease. At a time when epidemic scarlet fever visited Dresden, in 1831 and 1832, cases were seen of intense cerebral disorder without any eruption, differing from inflamation of the brain in the rapidity of attack and in the absence of many of the symptoms, and yet, by post-mortem examination, the conclusion was arrived at that these were cases of scarlatina, in which death had taken place before time was given for the manifestation of the disease. Sometimes a question arises whether the affection of the little sufferer is tubercular hydrocephalus or typhoid fever; but the character of the cerebral symptoms is different, and age also may often determine the diagnosis; the younger the child the less likely the disease to be hydrocephalus.

2d. "Those [symptoms] which at the outset of acute inflamations of the thoracic viscera sometimes throw the evidence of the real disease into the shade." Pneumonia and pleurisy, for instance, occasionally commence with a convulsion and vomiting, and delirium may follow, while the cough may be slight. Looking at the preponderance of severe cerebral symptoms over the evidences of thoracic disorder, cerebral congestion, hydrocephalus, or typhoid fever may be alike erroneously diagnosticated. But the physician cannot long remain in error, especially if he has the diagnostic skill to recognise the absence of many of the symptoms of diseased brain.

3d. "Those [symptoms] dependent on some disorder of the abdominal viscera," etc. Convulsions from such causes depend often on the indigestible character of the food supplied to the child, but the history of the case, the condition of the abdomen, and the peculiar character of the convulsion will generally establish the diagnosis. In diarrhœas, and relapses of diarrhœas, a condition may supervene, where the case has been persistent, in which irritation and exhaustion may produce symptoms like those of inflammation of the brain—hydrocephaloid disease, a kind of spurious hydrocephalus.

The same condition may arise from the gradual influence of imperfect nutrition, the cerebral symptoms coming on slowly and insiduously. As fatal results may follow this imitative disease as attend true inflammation of the brain, and yet if a treatment be adopted which aims at correcting the cause, instead of one founded upon a faulty diagnosis, such results may be averted.

4th. "Chronic cases, in which cerebral symptoms occur and are likely to be misapprehended." Dr. West briefly sketches a few of these, such as temporary but long continued dulling of the faculties after fevers, altered temper, a precociousness which becomes alarming from its unexpectedness, neuralgic headache, so often thought to be the precursors of mental diseases in children, etc.

In these warnings against the errors which may so readily occur in a hurried diagnosis, Dr. West has given valuable hints and suggestions of dangers which may attend the practice of physicians of any age in their care and management of the young sick.—*Medical Times and Gazette.*

